# CD8^+^ T-Cell Response to HIV Infection in the Era of Antiretroviral Therapy

**DOI:** 10.3389/fimmu.2019.01896

**Published:** 2019-08-09

**Authors:** Federico Perdomo-Celis, Natalia A. Taborda, Maria T. Rugeles

**Affiliations:** ^1^Grupo Inmunovirología, Facultad de Medicina, Universidad de Antioquia, Medellin, Colombia; ^2^Grupo de Investigaciones Biomédicas Uniremington, Programa de Medicina, Facultad de Ciencias de la Salud, Corporación Universitaria Remington, Medellin, Colombia

**Keywords:** CD8^+^ T-cell, HIV, antiretroviral therapy, IL-17, exhaustion, CXCR5

## Abstract

Although the combined antiretroviral therapy (cART) has decreased the deaths associated with the immune deficiency acquired syndrome (AIDS), non-AIDS conditions have emerged as an important cause of morbidity and mortality in HIV-infected patients under suppressive cART. Since these conditions are associated with a persistent inflammatory and immune activation state, major efforts are currently made to improve the immune reconstitution. CD8^+^ T-cells are critical in the natural and cART-induced control of viral replication; however, CD8^+^ T-cells are highly affected by the persistent immune activation and exhaustion state driven by the increased antigenic and inflammatory burden during HIV infection, inducing phenotypic and functional alterations, and hampering their antiviral response. Several CD8^+^ T-cell subsets, such as interleukin-17-producing and follicular CXCR5^+^ CD8^+^ T-cells, could play a particular role during HIV infection by promoting the gut barrier integrity, and exerting viral control in lymphoid follicles, respectively. Here, we discuss the role of CD8^+^ T-cells and some of their subpopulations during HIV infection in the context of cART-induced viral suppression, focusing on current challenges and alternatives for reaching complete reconstitution of CD8^+^ T-cells antiviral function. We also address the potential usefulness of CD8^+^ T-cell features to identify patients who will reach immune reconstitution or have a higher risk for developing non-AIDS conditions. Finally, we examine the therapeutic potential of CD8^+^ T-cells for HIV cure strategies.

## Epidemiology of HIV Infection and Impact of Antiretroviral Therapy

The human immunodeficiency virus type-1 (HIV) infection remains an important public health problem, particularly in developing countries. As a result of the long-term subclinical presentation of this infection, a high rate of shortfall at diagnosis is evidenced worldwide, and almost half of the HIV-infected patients are not aware of their HIV status (1). Therefore, most of the available data on HIV epidemiology is based on estimates, particularly in resource-limited settings (2). This issue dramatically affects public health policies; representing a major barrier for HIV global control. Thus, according to UNAIDS 2017 data, at the end of 2016, 36.7 million people lived with HIV, and of these, 17.8 million were women, and 2.1 million were children. The problem is increasing, as the global annual number of new HIV infections is 1.8 million, and deaths associated with the acquired immune deficiency syndrome (AIDS) reach 1 million.

The most important advance in the clinical management of HIV infection was the development of antiretroviral drugs and their therapeutic combination to suppress systemic viral load. Indeed, the combined antiretroviral therapy (cART), i.e., combination of three or more antiretroviral drugs, generally including at least two drugs of different mechanisms of action (1), has been effective in decreasing the deaths associated with AIDS. According to UNAIDS 2017 estimates, global AIDS-related deaths declined in 48% between 2005 and 2016, with near to eight million deaths prevented since the introduction of the therapy in 1995. Moreover, the decrease in the viral load by cART and consequent lower transmission risk, has led to the reduction of 16% of new HIV infections between 2010 and 2016. In addition, pre-exposure and post-exposure prophylaxis are recommended in some countries for prevention of HIV infection (3). Thus, cART constitutes a critical strategy for the control of HIV-associated morbidity and mortality, as well as a transmission prevention approach.

The World Health Organization (WHO) and Centers for Disease Control and Prevention (CDC) recommends the initiation of cART as soon as possible after diagnosis, resulting in improved viral control and prevention of AIDS conditions (available at https://www.who.int/hiv/topics/treatment/en/ and https://aidsinfo.nih.gov/guidelines). However, guidelines for management HIV-infected patients in some Latin American countries, such as Colombia, have clearly defined indications for initiation of cART, based on CD4^+^ T-cell counts and viral load (4). Importantly, from the total diagnosed patients in Colombia, 89.9% received at least one dose of antiretroviral drugs, although the number of patients receiving continuous therapy and with viral suppression (plasma or serum viral load <20 copies RNA/mL) only reached 66.6% of treated patients. This reflects the poor health system that is not able to sustain antiretroviral supply, in addition to problems with therapy adherence; these problems might be common in other Latin American countries. Overall, the strategies to increase the rate of diagnosis and the rapid initiation and maintenance of cART, will help to achieve the 90-90-90 target for HIV-infected patients (2).

## Current Challenges of the Antiretroviral Therapy

In addition to important issues in prevention, screening, and diagnosis of HIV-infection, the treatment of diagnosed patients has faced several challenges and pitfalls. These issues can be divided as following: (i) operative, related to coverage, adherence, and adequate monitoring of therapy; (ii) virological, related to generation of viral resistance to antiretroviral drugs, and (iii) immunological, related to immune reconstitution failure. Operative challenges include those associated with the lack of the required financial resources for implementing integral HIV programs; inadequate stocks of antiretroviral drugs; limited health system infrastructures; low acceptance of cART initiation and long-term adherence with absence of proven methods to ensure treatment adherence and an adequate follow-up. The complexity of cART regimens and the side effects of antiretroviral drugs are also a major complaint in HIV-infected patients. Moreover, the persistent stigma associated with HIV infection limits the timely detection of cases and early initiation of cART (5). The **second** type of cART challenge is the antiretroviral drugs resistance, which is the most frequent type of therapy failure, inducing a change in the first-line cART scheme (6). Drug resistance results from the high rate of viral mutations, enhanced by a poor cART adherence, pharmacokinetics limitations, inadequate dosing or drug interactions (7). In addition, a weak monitoring and limited indicators for antiretroviral drugs resistance reduce the rate of successful cART (8). Finally, partial immune reconstitution by cART is a major concern in the setting of viral suppression. It has recently attracted more attention, as it has been associated with increased non-AIDS conditions and related mortality, despite the reduction in AIDS-related deaths (9). Indeed, non-AIDS conditions, such as cardiovascular disease or stroke, are responsible for around 42% of deaths among HIV-infected patients with viral suppression induced by cART. Moreover, the mortality rate in these individuals is higher than in the general population, even excluding the AIDS-related conditions (10, 11).

Importantly, the success of cART in reducing AIDS-related deaths and increasing the life expectancy of HIV-infected patients, has resulted in a high number of patients over 50 years living with HIV (available at https://www.cdc.gov/hiv/group/age/olderamericans/index.html). Thus, the presence of concomitant diseases, such as metabolic or cardiovascular pathologies, and their respective medications, influence the choice of antiretroviral drugs for cART regimens, and increase the risk of side effects due to drug interactions (12).

## CD8^+^ T-Cells, Effector Functions, and Subpopulations

### Activation and Differentiation of CD8^+^ T-Cells

CD8^+^ T-cells are part of the adaptive immune system, playing a critical role for protection against foreign organisms and malignancies (13). The activation of naïve CD8^+^ T-cells requires three signals, provided by antigen-presenting cells (APC). Initially, there is the recognition of the antigen by the T-cell receptor (TCR); in the case of CD8^+^ T-cells, the peptides are 8–10 amino acids in length and are presented by major histocompatibility complex (MHC) class I molecules. Second, costimulatory signals are required, such as the binding of CD80 or CD86 from the APC to the CD28 molecule expressed by the CD8^+^ T-cell, and finally an alarm signal produced in response to pathogens, such as IL-12 and type I interferon (IFN), among others (14, 15).

After activation, CD8^+^ T-cells undergo clonal expansion, generating a large pool of effector cells. These cells exhibit high effector functions, such as the expression of cytokines, cytotoxic molecules and a high capacity for degranulation (16–19). Additionally, at this point, effector CD8^+^ T-cells acquire the ability to migrate to peripheral tissues (20). After clonal expansion, effector cells suffer massive apoptosis, in a period known as contraction phase. Finally, the remaining antigen-specific CD8^+^ T-cells constitute the pool of memory cells, which decrease their effector profile, remaining in a quiescent state expecting a new antigenic challenge. This period, which can last the whole life of the individual, is known as the memory phase (21). Several CD8^+^ T-cells differentiation models propose that memory cells can differentiate into effector cells once they are exposed to activation signals, acquiring a high capacity for cytokine production and cytotoxic potential (22).

### CD8^+^ T-Cells Effector Mechanisms

**Figure 1:**
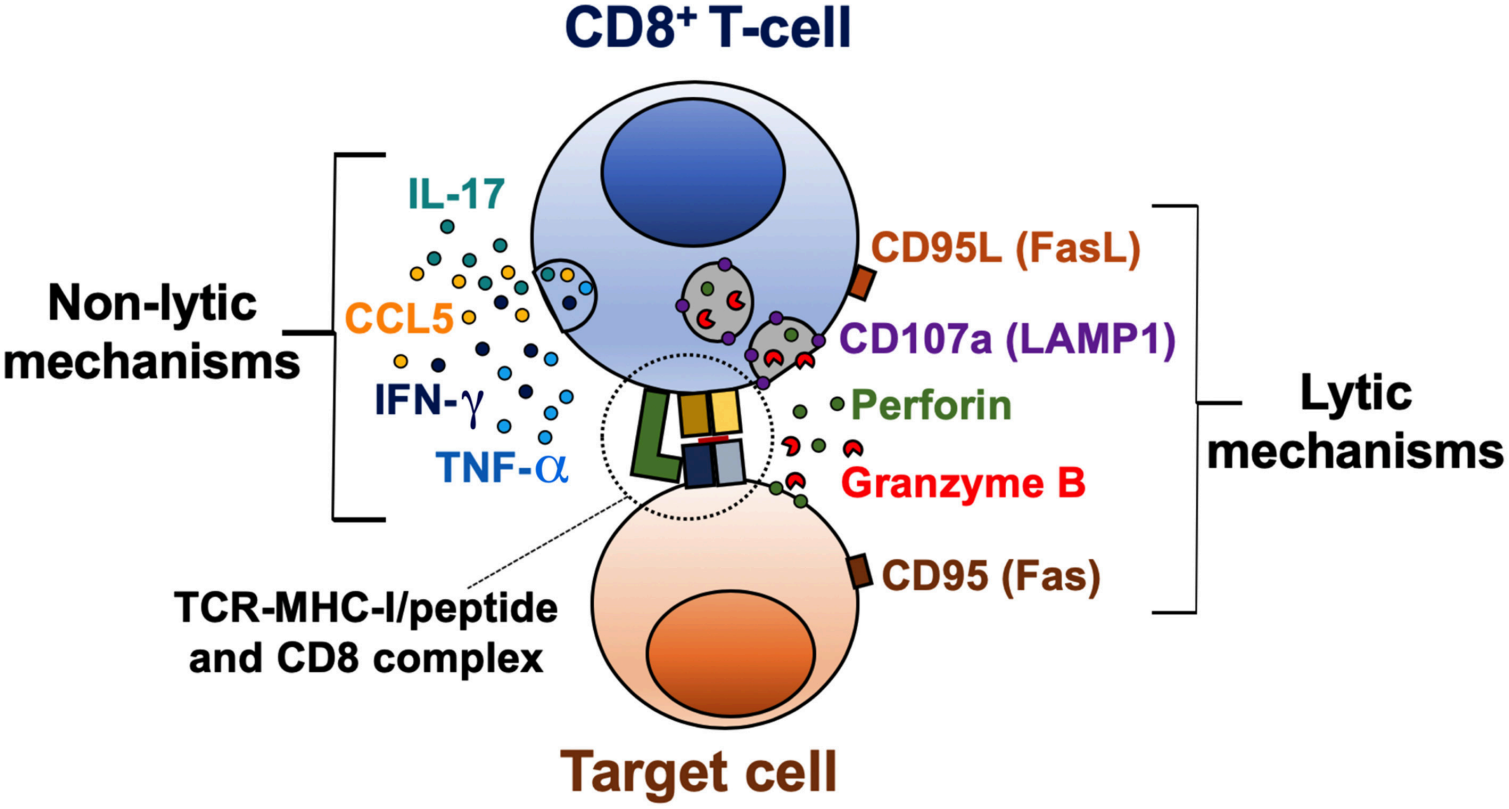
Lytic and non-lytic effector mechanisms of CD8^+^ T-cells. CD8^+^ T-cells are activated after recognition of an MHC-I/peptide complex, which binds to the TCR and CD8 molecules. The cytotoxic potential of CD8^+^ T-cells is determined by the expression of cytotoxic molecules granzyme B and perforin, and a coordinated degranulation process, which can be evaluated by the cell membrane expression of CD107a (LAMP1). Receptor-mediated cell death via CD95L/CD95 interaction is also a lytic mechanism of CD8^+^ T-cells. Activated CD8^+^ T-cells can also produce cytokines such as IFN-γ, TNF-α, and IL-17, and the β-chemokine CCL5, which exert a variety of antiviral, inflammatory, and regulatory functions.

CD8^+^ T-cell effector mechanisms can be classified in lytic (cytotoxicity) and non-lytic (cytokine production) (Figure 1). These effector functions are primarily regulated by the balance between the T-bet, Eomes, and Runx families of transcription factors (23–26). Indeed, these transcription factors not only bind to DNA sequences of effector molecules such as granzyme B and IFN-γ, but also cooperate with chromatin remodeling proteins to regulate chromatin accessibility of key genes in activated CD8^+^ T-cells (27). The cytotoxic capacity of CD8^+^ T-cells depends on the content of their lytic granules and their degranulation capacity (28, 29). Lytic granules are secretory lysosomes containing effector molecules, such as granzymes and perforin (30). The core of the granule is surrounded by a lipid bilayer containing lysosome-associated membrane glycoproteins (LAMP), including LAMP-1 (CD107a), LAMP-2 (CD107b), and LAMP-3 (CD63) (31). During the degranulation process, the granule membrane is fused with the plasma membrane of the activated CD8^+^ T-cell, and the content of the granule is released into the immunological synapse between the CD8^+^ T-cell and the target cell (29). Of note, LAMP molecules are not found on the surface of resting CD8^+^ T-cells. Therefore, the evaluation of the surface expression of CD107a/b allows to identify the degranulation of CD8^+^ T-cells (28, 29). In addition, lytic granules also contain the membrane pore-forming protein granulysin (32), the proteoglycan matrix protein serglycin (33), the perforin inhibitor calreticulin (34), and the lysosomal enzymes cathepsins (35). Moreover, apoptosis-inducing Fas ligand (CD95L) is stored in specialized secretory lysosomes of CD8^+^ T-cells and the degranulation process controls its expression at the cell surface (36).

Perforin and granzymes are the most abundant proteins within the lytic granules of CD8^+^ T-cells and cooperate for inducing death of target cells (37). Apparently, granzymes work in a perforin-dependent manner, since initial studies in mast cell lines, the presence of perforin was required for granzyme B-induced death of target cells (38). Thus, the pore formation by perforin facilitates the entry of granzymes into target cells (39). However, granzymes could be internalized by endocytosis, after binding to the mannose-6-phosphate receptor in target cells (40). Molecular effector mechanisms can vary among granzyme subtypes, inducing apoptosis through caspase-dependent and independent mechanisms (41). Once in the cytosol of target cells, granzymes cleave the BH3-interacting domain death agonist (BID) and pro-caspase 3 (42, 43). Truncated BID alters the mitochondrial membrane, inducing the release of pro-apoptotic factors such as cytochrome C, involved in the formation of the apoptosome (44), and endonuclease G, which causes DNA fragmentation (45). In addition, active caspase 3 also induces endonuclease and protease activation, causing degradation of cellular DNA and cytoskeletal proteins (46).

Cytokines secreted by CD8^+^ T-cells include IFN-γ, TNF-α, and IL-2, important for promoting antiviral, inflammatory responses, and T-cell survival and proliferation, respectively (47). Additionally, CD8^+^ T-cells secrete the β-chemokines macrophage inflammatory protein (MIP)-1α (CCL3), MIP-1β (CCL4), and regulated upon activation, normal T-cell expressed and secreted (RANTES; CCL5). These chemokines bind to the receptors CCR1, CCR3, CCR4, and CCR5, some of them, previously characterized as HIV co-receptors (48); therefore they play an important role in this infection by blocking viral binding and entry into target cells (49, 50).

### CD8^+^ T-Cell Subpopulations

Several CD8^+^ T-cell subsets have been described, based on the expression of the differentiation markers CD45RA, CD45RO, CCR7, CD62L, CD27, and CD28, among others. The most extended classification divides them into naïve, central memory, effector memory, and terminal effector cells; each of them has different effector capacities, particularly evaluated by the expression of granzymes, perforin, IFN-γ, the degranulation ability, and *in vitro* cytotoxicity [Table 1; (18, 51)]. The lowest functional capacity is observed for naïve CD8^+^ T-cells, which have low expression of effector molecules, reduced degranulation capacity and low *in vitro* cytotoxicity. Central memory cells have a low/intermediate cytotoxic potential, given their low basal expression of granzymes and perforin, which confers them a limited immediate *in vitro* cytotoxicity. However, these cells can degranulate and express *de novo* effector molecules after polyclonal or antigen-specific stimulation (22, 52). Finally, effector memory and terminal effector cells are characterized by a high cytotoxic capacity (Table 1).

**Table 1:** Effector capacity of CD8^+^ T-cells according to their differentiation stage.

**Cell subset**	**Phenotype**	**Degranulation (CD107a/b expression)**	**Cytotoxic molecules expression (granzymes and/or perforin)**	***In vitro* cytotoxicity**	*De novo* synthesis of granzymes and/or perforin^*^	IFN-γ production^*^	**Main location**
Naïve	CD45RA^+^ CD45RO^−^ CCR7^+^ CD62L^+^ CD28^+^ CD27^+^ CD57^−^	–	–	–	–	–	Secondary lymphoid tissues
Central memory	CD45RA^−^ CD45RO^+^ CCR7^+^ CD62L^+^ CD28^+^ CD27^+^ CD57^−^	++	++	++^*^	++	+++	Secondary lymphoid tissues
Effector memory	CD45RA^−^ CD45RO^+^ CCR7^−^ CD62L^−^ CD28^+/−^CD27^+/−^CD57^−/+^	+++	+++	+++	+	+++	Blood and inflamed tissues
Terminal effector	CD45RA^+^ CD45RO^−^ CCR7^−^ CD62L^−^ CD28^−^ CD27^−^ CD57^+^	+++	+++	+++	+	+++	Blood and inflamed tissues

* *After 5 h stimulation*.

Of note, the distribution of CD8^+^ T-cell subsets and their effector machinery also vary according to the body tissues and compartments, with effector CD8^+^ T-cells mainly located in blood and inflamed tissues, and naïve and central memory cells primarily found in secondary lymphoid organs (Table 1). Particularly, their transcriptional program determines this cell distribution and effector function. Thus, CD8^+^ T-cells in lymphoid tissues, as well as in gastrointestinal mucosa, from healthy individuals, have a lower expression of perforin and granzyme B compared with blood cells, which is associated with a low expression of T-bet in these tissues (53–55). Interestingly, CD8^+^ T-cells in lymphoid tissues are poised to upregulate cytotoxic molecules and rapidly exert effector functions, similar to long-lived memory CD8^+^ T-cells (22). However, they also upregulate trafficking markers such as CXCR3 to egress from lymphoid tissues (53). Moreover, compared with blood cells, memory CD8^+^ T-cells from tonsil, lymph nodes, and spleen exhibit higher tissue residency markers such as CD69, CD103, and CD49a, but lower expression of T-bet, eomes, perforin, and granzyme B. Therefore, cells from blood and tissue compartment form distinct phenotypic and functional clusters (56).

According to the cytokines they produce, several subpopulations of CD8^+^ T-cells have been described. Similar to CD4^+^ T-cells, IFN-γ-producing, and IL-5/IL-13-producing CD8^+^ T-cells are designated as Tc1 and Tc2 cells, respectively (57). Regulatory CD8^+^ T-cell subsets have also been described (58). Moreover, IL-17-producing CD8^+^ T-cells (Tc17) can be induced by polarizing cytokines such as Transforming Growth Factor (TGF)-β1 and IL-6 (59). This population is characterized by the expression of the C-type lectin receptor CD161 (60), the transcription factor retinoic acid receptor-related orphan nuclear receptor (ROR)-γt (61), and its main localization is the intestinal tract (62–64). Importantly, the IL-17 cytokine family is constituted by six proteins that share homology (IL-17A through IL-17F) (65). The most widely studied is IL-17A (here referred as IL-17), and pro-inflammatory and immunomodulatory effects have been described (66). In the context of HIV infection, this cytokine has attracted attention due to its beneficial effects in the gut mucosa, such as the promotion of the conformation of tight junctions in epithelial cells (67), secretion of antimicrobial peptides (68), as well as recruitment of immune cells to sites of mucosal injury (69). In this sense, Tc17 cells could promote gut homeostasis, exerting a protective mechanism during HIV infection. Finally, similar to CD4^+^ T-cells, follicular CXCR5-expressing CD8^+^ T-cells have been characterized (70), and their role during HIV infection is examined below.

## Progressive Dysfunction of CD8^+^ T-Cells During HIV Infection and Poor Reconstitution Despite cART

CD8^+^ T-cells are important in the control of HIV replication (71), as evidenced by: (i) emergence of HIV-specific CD8^+^ T-cells coinciding with the decrease of viremia in acutely infected patients (72); (ii) a potent HIV-specific CD8^+^ T cell response contribute to the reduction of the pool of HIV-infected cells and the HIV reservoir (73); (iii) increase in viral load in SIV-infected macaques after CD8^+^ T-cell depletion (74, 75); (iii) associations between the frequency and/or functional capacity of HIV-specific CD8^+^ T-cells and limited viral replication and/or disease non-progression in HIV-infected patients (76, 77); (iv) appearance of viral escape mutations to evade the immune pressure of HIV-specific CD8^+^ T-cells (78); (v) requirement of CD8^+^ T-cells for maintaining therapy-induced viral suppression in SIV-infected macaques (79). In fact, some CD8^+^ T-cells features have been proposed as correlate of protection in HIV-infected patients (80).

While HIV-specific CD8^+^ T-cells control the virus during acute HIV/SIV infection, their cytotoxic potential dramatically decreases along with disease progression and are no longer capable of exerting an appropriate antiviral response (81, 82). Certainly, CD8^+^ T-cells suffer important alterations in their frequency, differentiation, and activation profile, undergoing immune exhaustion, and progressive dysfunction (24, 83–87). Compared with seronegative individuals, the total pool of circulating CD8^+^ T-cells is persistently increased in untreated HIV-infected patients (88), with higher frequency of memory subsets and reduction of naïve cells (89, 90). In addition, patients exhibit higher expression of the activation markers HLA-DR, CD38, and Ki-67 (91), and immune exhaustion markers such as programmed death (PD)-1 and T-cell immunoglobulin and mucin-domain containing-3 (TIM-3) (92). Remarkably, the HLA-DR^+^ CD38^+^ Ki-67^+^ PD-1^+^ phenotype in CD8^+^ T-cells characterizes the effector phase after acute viral infections or vaccination, which is associated with disease control (16, 93–96). Nonetheless, the expression of these molecules during chronic HIV infection is accompanied by the impairment of CD8^+^ T-cell lytic and non-lytic mechanisms (24, 83, 97), as well as the proliferative ability and survival (98–100). Specific subpopulations, such as Tc17 cells, are decreased in gut mucosa and blood during HIV/SIV infections (101–103). Importantly, these alterations are observed in HIV-specific CD8^+^ T-cells (76, 104), as well as cells specific for other pathogens, such as cytomegalovirus (CMV), Epstein-Barr virus, influenza virus, and adenovirus (105, 106). Taking into account that HIV-specific cells constitute <20% of the total CD8^+^ T-cell population (107, 108), these data reflect the massive bystander activation that CD8^+^ T-cells undergo during HIV infection.

Considering that a large size of viral burden is a product of infected cells in lymph nodes and gastrointestinal mucosa (109), and there is a low distribution of antiretroviral drugs to these compartments (110), an effective CD8^+^ T-cell response in these tissues is required to control viral replication and the reservoir size. Nonetheless, the cytolytic activity of lymph node CD8^+^ T-cells is reduced in chronically HIV-infected patients (both untreated and on cART) compared with seronegative controls (53). Furthermore, lymph node and rectal HIV-specific CD8^+^ T-cells have lower expression of granzyme B and perforin, as well as T-bet, compared with blood cells (53, 55). Thus, while there is a regulated cytolytic function in CD8^+^ T-cells in lymphoid tissues and gut mucosa, there is apparently a poorer cytotoxic response in HIV-infected patients. Intriguingly, HIV controllers maintain low viral load in the absence of therapy and despite this low cytotoxic potential of CD8^+^ T-cells in lymphoid tissues (53, 111). Thus, it is possible that non-lytic mechanisms and a polyfunctional response, including IFN-γ, TNF-α, and MIP-1β production, as well as degranulation, are critical in the control of HIV infection in lymphoid and gastrointestinal tissues (111, 112).

Along with the suppression of viral load and the increase in the CD4^+^ T-cell counts, cART induces improvement of some of the CD8^+^ T-cell alterations found in HIV-infected patients (113–115), whereas treatment discontinuation causes the increase of CD8^+^ T-cell activation and dysfunction (116, 117). However, compared with seronegative individuals, cART does not fully reconstitute the CD8^+^ T-cell counts (88, 118), the proportions of memory subsets, the levels of activation and exhaustion markers (91, 118–121), and their functional capacity (24, 122). In addition, the loss of Tc17 cells and CD161-expressing CD8^+^ T-cells is not restored in HIV-infected patients under suppressive cART (101, 102, 120). Interestingly, in seronegative individuals, HLA-DR^+^ CD38^+^ cells constitute the main IL-17-producing subset among CD8^+^ T-cells (120), consistent with an effector memory profile of HLA-DR-expressing CD8^+^ T-cells (123). However, this subset is decreased in HIV-infected patients on cART (120). Remarkably, early initiation of cART is associated with improved reconstitution of CD8^+^ T-cell counts and activation levels (124, 125), whereas a long treatment is required for improvement of some CD8^+^ T-cell phenotypic and functional disturbances (120, 123).

It is important to mention that the decrease in antigen load with ART induces a decline in the frequency of circulating HIV-specific CD8^+^ T-cells (108, 115, 126, 127), with subsequent increases after treatment interruption or failure (108, 127, 128). Changes in the frequencies of circulating CMV-specific CD8^+^ T-cells are also observed in patients with treatment interruption (108, 115, 127). HIV-specific CD8^+^ T-cells are maintained in lymph nodes (128) and exert potent antiviral responses in *ex vivo* assays (129), but are not able to suppress viral replication in the absence of therapy. This issue represents a major challenge in the setting of supervised treatment interruptions strategies in the search of HIV cure strategies (130). Certainly, memory virus-specific CD8^+^ T-cells respond to the changes in antigen load and the inflammatory milieu during cART, actively migrating within body compartments and possibly modulating their effector response.

## Causes of CD8^+^ T-Cell Dysfunction During HIV Infection

Three factors are major determinants of CD8^+^ T-cell dysfunction during chronic infections (131): persistent antigen, negative costimulation, and chronic inflammation. In addition, the loss of CD4^+^ T-cell help enhances CD8^+^ T-cell dysfunction during HIV infection (132), whereas cell-intrinsic defects may also contribute to this pathogenic process (133). Remarkably, even in the context of cART-induced viral suppression, the three major determinants of CD8^+^ T-cells dysfunction are present, since there is a residual HIV replication and microbial burden that maintain a persistent antigen load (110, 134, 135), induce continuous expression of inhibitory receptors (131), and the secretion of inflammatory mediators (136–138).

### Persistent Antigen and Chronic Inflammation

Chronic immune activation is a hallmark of HIV infection (139), and it is associated with phenotypic and functional changes of immune cell populations (140), impairment of antiviral mechanisms (141), increase in the number of target cells (142), CD4^+^ T-cell regenerative failure (143, 144), and risk of organ damage (145). HIV-associated immune activation is explained by the persistent viral replication and reactivation of HIV reservoirs, recurrence of co-infections, loss of the integrity of the gut mucosa, and increased systemic levels of pro-inflammatory cytokines [such as IL-6, IL-1β, and tumor necrosis factor (TNF)-α], among other factors (139). Of note, the maintenance of low immune activation levels characterizes non-pathogenic simian immunodeficiency virus (SIV) infection in natural hosts despite sustained viral replication (146). Moreover, the levels of immune activation predict the magnitude of CD4^+^ T-cell depletion better than viral loads in HIV-infected patients (91, 147), and are associated with disease progression, the development of AIDS-defining and non-AIDS conditions, and mortality (148–150).

Tissue reservoirs (i.e., tissues containing cells with integrated HIV) promote a persistent antigenic burden during HIV infection, even during cART. In a macaque model of SIV infection, the primary reservoir sites of infection were lymphoid tissues (~98% of total RNA^+^ cells), including lymph nodes, spleen, and gut-associated lymphoid tissues (GALT) (109). Other tissues, such as brain, kidney, heart or lung, individually contributed to <1% of RNA^+^ cells (109). Similarly HIV tissue reservoirs are predominant in GALT (151), and lymph nodes (152). In lymphoid follicles, CD4^+^ T-cells, particularly of the CXCR5^+^ follicular subset, exhibit high levels of infection (153), and free virions are captured by follicular dendritic cells (154). Remarkably, although cART decreases the HIV reservoir size in lymph nodes, HIV RNA and DNA can still be detected after years of therapy (152, 155), whereas there is minimal cART-induced change in HIV DNA in gut tissues (109). Thus, viral reservoirs constitute an important source of persistent antigen, even during cART.

**Figure 2:**
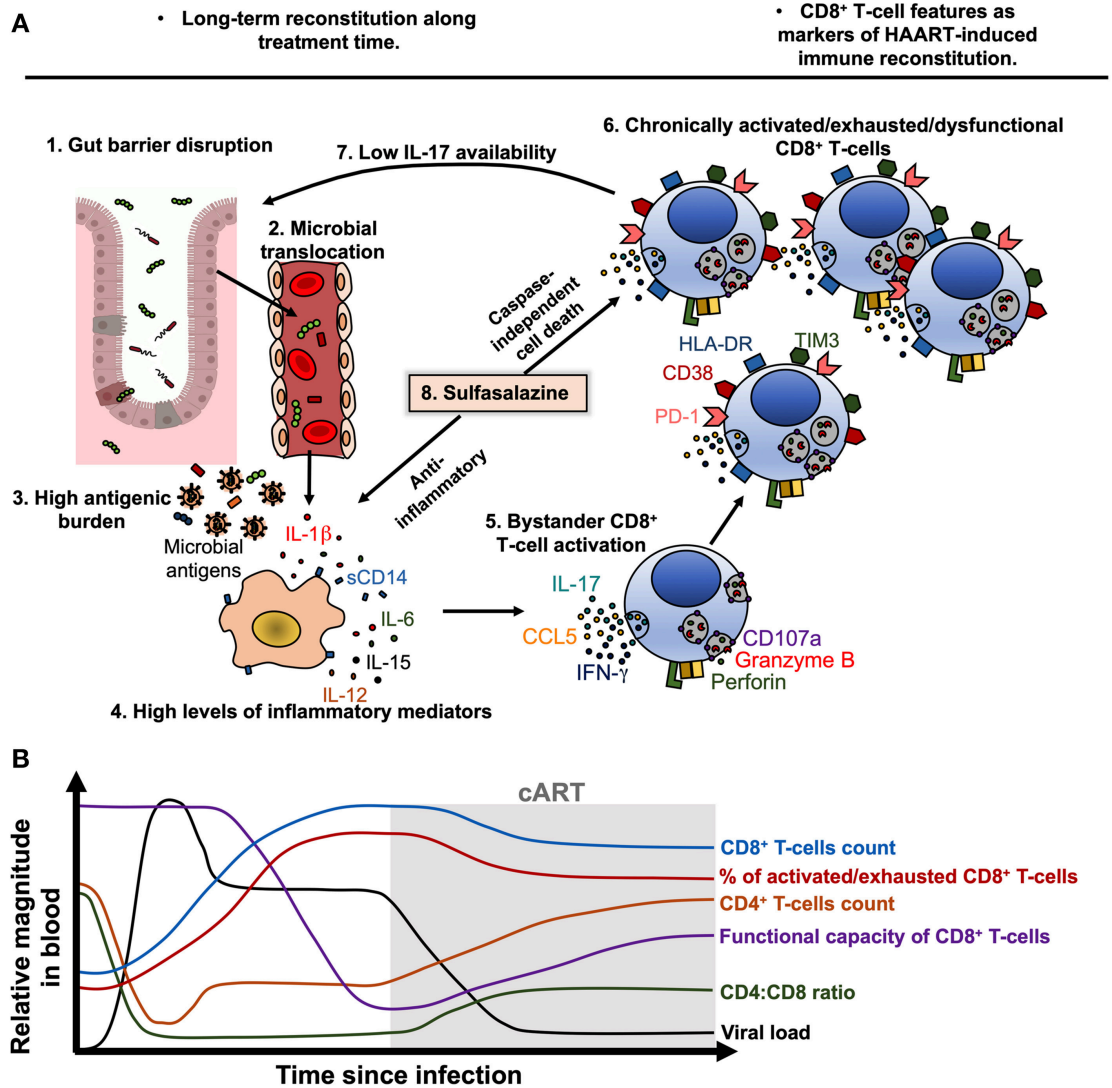
Model of CD8^+^ T-cell activation, exhaustion and dysfunction during treated HIV infection. **(A)** During chronic HIV, and despite cART-induced viral suppression, gut barrier disruption (1) causes the passage of microbial products to systemic circulation (microbial translocation, 2), contributing to a high antigenic burden together with residual HIV replication and ongoing co-infections (3). Microbial products and other antigens activate innate immune cells such as monocytes/macrophages, which activate and release soluble CD14 (sCD14) and inflammatory cytokines such as IL-1β, IL-6, IL-15, and IL-12 (4). Particularly, IL-15 and IL-12 induce bystander activation of CD8^+^ T-cells (5), which in early stages of disease may exhibit potent cytotoxic capacity and cytokine production. Nonetheless, chronic bystander stimulation induces exhaustion and dysfunction of CD8^+^ T-cells (6), which have an increased expression of HLA-DR, CD38, PD-1 and TIM3, and lower degranulation capacity and IL-17 secretion, among other alterations. The low secretion of IL-17 causes a low availability of this cytokine and its beneficial effects on gut mucosa, worsening the gut barrier disruption (7). In this setting, sulfasalazine could be a therapeutic approach to target the inflammatory environment and activated/dysfunctional CD8^+^ T-cells, through the inhibition of inflammatory cytokine secretion and induction of caspase-independent cell death (8). **(B)** Model of the dynamic of viral load, CD4^+^ T-cells count, CD8^+^ T-cells count, CD4:CD8 ratio, the frequency of activated/exhausted CD8^+^ T-cells and their functional capacity in HIV-infected patients before and after ART beginning. Of note, the viral load is efficiently suppressed and the CD4^+^ T-cells count are recovered after treatment beginning, but the CD8^+^ T-cells features remain disturbed despite therapy.

The loss of the integrity of the gut mucosa is also responsible for a high antigenic burden that consequently drives immune dysfunction (156). Mechanistically, this process can be viewed as follows (Figure 2A):

1. Alteration in the gut mucosa: during HIV/SIV infection, it is induced by the decrease of mucosal IL-17/IL-22-producing cells (157, 158), loss of gut junctional complexes (159, 160), changes in the microbiota composition (161), and persistence of HIV reservoirs in GALT (151).
2. Microbial translocation: Gut barrier disruption allows the passage of microbial products from the intestinal lumen to the lamina propria and systemic circulation (162).
3. Activation of immune cells by microbial-associated molecular patterns (MAPS) via pathogen-recognition receptors (PRRs): innate immune cells are activated by microbial components via PRRs, such as Toll-like receptors (TLR) (163, 164). Importantly, in chronic inflammatory settings (165, 166), as well as during HIV infection (167), CD4^+^, and CD8^+^ T-cells may upregulate TLR-2, 3, 4, 7, and 9 expression (167, 168), and human T-cells can respond *in vitro* to several MAPS (168, 169). In the case of CD4^+^ T-cells, this TLR-mediated activation renders them more susceptible to HIV replication (170) and apoptosis (169), whereas in the case of CD8^+^ T cells, TLR engagement lowers the activation threshold (171), which can be deleterious in a chronic setting. Thus, T-cell exposure to TLR agonists may directly contribute to increased T-cell activation during chronic HIV infection.
4. Cytokine secretion and immune cells activation and dysfunction: IL-1β, IL-18, IL-6, TNF-α, and type-I IFN induced by PRRs ligation promote the activation of innate and adaptive immune cells (172, 173). Moreover, cytokines such as IL-15 and IL-12 are an important signal for bystander activation of CD8^+^ T-cells, particularly memory subsets (105).

### Negative Costimulation

As previously mentioned, CD8^+^ T-cells from HIV-infected patients exhibit increased expression of inhibitory receptors such as PD-1, TIM-3, lymphocyte Activation Gene-3 (LAG-3), CD160, and 2B4 (86, 120, 174). Overall, these receptors interfere with TCR signaling by competing with costimulatory ligands, modulating intracellular pathways, or inducing inhibitory molecules in T-cells; these mechanisms decrease the response of antigen-specific cells to cognate stimulation (175). Expression of inhibitory receptors, particularly PD-1, is also associated with a reduced functional capacity of total CD8^+^ T-cells after polyclonal stimulation, such as lower degranulation capacity, with consequent decreased release of the cytotoxic molecules granzyme B and perforin (123), and a lower proportion of IL-17-producing cells [(120); Figure 2A]. Moreover, activated/exhausted CD8^+^ T-cells present higher susceptibility to apoptosis (104, 176, 177), as in the case of Tc17 cells, which are highly activated and exhausted during HIV infection and are susceptible to activation-induced cell death (102, 178). Finally, cell-intrinsic defects, such as an impaired signaling machinery (133), and an altered transcriptional, epigenetic, and metabolic profile may also account for the observed dysfunction in activated/exhausted CD8^+^ T-cells during HIV infection (24, 179, 180).

## Potential Immunomodulatory Strategies for Improving CD8^+^ T-Cells Immune Reconstitution During Antiretroviral Therapy

### cART Intensification

Some studies indicate that the intensification of the cART with HIV integrase inhibitors, such as raltegrevir promotes the normalization of the level of activation of CD8^+^ T-cells (181, 182). Indeed, according with their mechanism of action, integrase inhibitors decrease the levels of proviral DNA (182–185), impacting in the reservoir size and the consequent antigenic burden and immune activation. The effect of raltegravir intensification of cART is also evidenced by the decrease in the levels of the coagulation marker D-dimer (184).

### Anti-inflammatory Agents

Since chronic immune activation and systemic inflammation are important determinants of HIV-associated morbidity and mortality, some anti-inflammatory drugs have been explored in the setting of HIV infection, including acetylsalicylic acid, statins, and hydroxychloroquine [reviewed in (186)]. Among them, the treatment with the statins atorvastatin or rosuvastatin in the presence of cART has shown a reduction in the levels of HLA-DR/CD38 and/or PD-1-expressing CD8^+^ T-cells compared with placebo controls (187–189). We also explored the effect of sulfasalazine (SSZ) on the reconstitution of CD8^+^ T-cells functional capacity, focused on IL-17 production (120). Sulfasalazine molecule combines the antibiotic sulphapyridine with the anti-inflammatory drug 5-aminosalicylic acid, and has been used in the treatment of chronic inflammatory diseases (190). Previous reports indicated that SSZ not only inhibits macrophage activation and secretion of TNF-α (191, 192), but also induces apoptosis of activated—and possibly dysfunctional—T-cells (193). Certainly, *in vitro* analyses with cells derived from HIV-infected patients on cART, showed that SSZ induces caspase-independent cell death of CD8^+^ T-cells co-expressing HLA-DR and CD38 (120), possibly through the mitochondrio-nuclear translocation of the apoptosis- inducing factor (AIF), that induces DNA fragmentation (193). Moreover, SSZ decreased the levels of LPS-induced IL-1β. These mechanisms were associated with an increase in the proportion of Tc17 cells in HIV-infected patients [(120); Figure 2A]. Interestingly, clinical observations from Colombian health care programs for HIV indicate that SSZ improves the symptoms of HIV-infected patients suffering wasting syndrome. This effect could be related with the abnormal T-cell activation in this group of individuals (194), that is targeted by SSZ. Thus, the use of SSZ and/or other anti-inflammatory drugs could be considered for managing patients with poor response to cART or with advance/progressive disease; large clinical studies that address its usefulness are needed.

### Cytokines

Due to the high relevance of Tc17 cells in the context of HIV infection, previous studies have evaluated different strategies to reconstitute this population, as well as mucosa-associated invariant T (MAIT) cells, which are an important IL-17-producing CD161^hi^ CD8^+^ subset. The administration of IL-7, a member of the γ-common chain family of cytokines, to HIV-infected patients on cART increases the frequency and number of circulating MAIT cells (195). *In vitro*, IL-7 also promotes the cytotoxic capacity and cytokine production of MAIT cells (196). This approach could be relevant in the clinical setting to limit the deterioration of the gut barrier, most likely preventing microbial co-infections. Another member of the γ-common chain, IL-21, promotes the survival, expansion, and cytotoxic responses of antigen-specific CD8^+^ T-cells (197–199). Accordingly, the administration of IL-21 to SIV-infected rhesus macaques increased the expression of cytotoxic molecules and polyfunctional CD8^+^ T-cells, compared with untreated controls (200). A functionally-related cytokine, IL-15, also increased the levels of granzyme B^+^ and perforin^+^ CD8^+^ T-cells after administration (in the form of complex between IL-15 and IL-15 receptor α) in uninfected and SHIV-infected rhesus macaques, both in cells from peripheral blood, lymph nodes, and mucosa (201). Interestingly, IL-15 treatment also promoted the migration of cytotoxic CD8^+^ T-cells to lymphoid follicles (201), whereas administration of an IL-15 superagonist to SIV-infected macaques induced this effect through the induction of the expression of the follicle-homing chemokine receptor CXCR5 in CD8^+^ T-cells (202). Thus, γ-common chain cytokines are potentially useful as immunotherapies for promoting CD8^+^ T-cells function HIV-infected patients, and clinical studies are required to evaluate their effectiveness.

### Checkpoint Therapy

Checkpoint therapy (i.e., blockade of the aforementioned inhibitory receptors or their ligands) may constitute an important strategy to improve CD8^+^ T-cell function in HIV-infected patients on cART. Certainly, *in vitro* blockade of PD-1 or its ligands PD-L1/L2 promotes HIV or SIV-specific CD8^+^ T-cells function and proliferative capacity (83, 203). Similarly, evidence in macaque models of SIV infection indicates that anti-PD-1 antibody therapy increases the frequencies of SIV-specific CD8^+^ T-cells in blood and gut, with improved polyfunctional capacity and proliferative potential; this effect is associated with significant reductions in viral load and increased survival of infected macaques (204). Interestingly, PD-1 blockade also promotes the response of CD8^+^ T-cells against gut-resident pathogens, as well as contribute to reduce gut epithelial damage, microbial translocation, and immune activation (205). Moreover, the administration of anti-PD-1 antibody to rhesus macaques prior initiation of cART enhanced the antiviral function of CD8^+^ T-cells, whereas promoted the expansion of CXCR5^+^ perforin^+^ granzyme B^+^ CD8^+^ T-cells after cART interruption, resulting in a better control of viremia (206). Interestingly, the CXCR5^+^ subset is the main CD8^+^ T-cell population responding to PD-1 blockade (207), which would be important in the context of HIV infection due to the role of follicles as tissue reservoirs (discussed below). Another effect of PD-1 axis blockade would be the reversal of HIV latency (208), that could contribute to recognition and elimination of infected cells by functionally improved HIV-specific CD8^+^ T-cells. In line with this evidence, a phase I clinical trial of an anti-PD-L1 antibody in 8 HIV-infected patients on cART showed that the frequency of Gag-specific CD8^+^ T-cells expressing IFN-γ or CD107a increased from baseline to day 28 post-treatment, although there was no statistical significance, most likely due to high inter-individual variability. There were no changes in the CD4^+^ T-cell counts or CD4:CD8 ratio (209). Importantly, an overall safety has been shown for the anti-PD-L1 or anti-PD1 antibody therapy, even in patients with concomitant malignancies (209, 210). Together, these studies indicate that PD-1 axis blockade could improve the antiviral function of CD8^+^ T-cells during HIV infection, along with other beneficial effects on the levels of microbial translocation, immune activation and viral reservoirs.

## CD8^+^ T-Cells as Correlates of Immune Reconstitution During Antiretroviral Therapy

A large body of evidence indicate that monitoring CD4^+^ T-cell counts, and viral load is informative of the effectiveness of cART-induced viral suppression in HIV-infected patients. Typically, CD4^+^ T-cells increase rapidly in the first weeks of treatment, followed by a more gradual increase, depending on the level of nadir CD4^+^ T-cells or the naïve subpopulation at the time of treatment initiation (211–215). Moreover, HIV-infected patients who maintain viral suppression and had CD4^+^ T-cells ≥300 cells/μL are unlikely to experience counts <200 cells/μL (threshold for opportunistic infection risk), suggesting that routine CD4^+^ T-cells monitoring may be unnecessary in some scenarios (216). Certainly, undetectable viral load or CD4^+^ T-cell counts do not reflect the immunological alterations that are present in treated HIV-infected patients. In contrast, the persistently high CD8^+^ T-cell counts and low CD4:CD8 ratio are indicative of partial immune reconstitution in treated HIV-infected patients, and, due to their accessibility in low-income settings and predictive power of adverse clinical outcomes (88, 217, 218), could be useful tools for immune monitoring of these individuals. Moreover, in HIV-infected patients under long-term suppressive cART (more than 25 months) there is a recovery in the proportion of CD107a^+^ and IL-17^+^ CD8^+^ T-cells, reaching similar levels to those seen in seronegative individuals (120, 123). These findings suggest a potential usefulness of these CD8^+^ T-cell functional markers to predict immune reconstitution in HIV-infected patients on cART, in addition to others currently used such as the CD8^+^ T-cell counts and the CD4:CD8 ratio (88, 217, 218).

In summary, cART-induced immune reconstitution is a long-term and incomplete process. After treatment initiation, viral load is rapidly controlled and CD4^+^ T-cell counts are progressively recovered. However, CD8^+^ T-cell counts remain increased despite cART, along with a low CD4:CD8 ratio (Figure 2B). Moreover, the frequency of activated/exhausted CD8^+^ T-cells do not completely return to basal levels, and some grade of dysfunction remains in several CD8^+^ T-cell subsets (Figure 2B). The improvement of cART effectiveness may require the combination of strategies such as an early beginning of therapy, rigorous clinical monitoring, the use of reliable biomarkers and possibly the use of immunomodulatory therapies.

## CXCR5^+^ CD8^+^ T-Cells and Their Role During HIV Infection

Another subpopulation of CD8^+^ T-cells that is relevant in the setting of HIV infection is that expressing CXCR5, since they could be a potential therapeutic cell-based strategy to eradicate HIV in follicles (219), an important body viral reservoir (153, 220). CXCR5-expressing CD8^+^ T-cells follow a particular dynamic during HIV/SIV infection. A common observation across studies is the increase in the proportion of CXCR5^+^ CD8^+^ T-cells in lymphoid follicles in HIV-infected patients, compared with seronegative controls (70, 221–223). A similar accumulation of follicular CXCR5^+^ CD8^+^ T-cells has been observed in SIV-infected macaques (224, 225). This accumulation is associated with the inflammatory/immune activation environment within follicles and increased levels of the CXCR5-ligand CXCL13 in HIV-infected patients, but not with the levels of viral replication or antigens, both during HIV (222) and SIV infection (225). Moreover, CXCR5^+^ CD8^+^ T-cells could also migrate within lymph nodes in response to CXCL10, which is ligand of CXCR3, as has been observed in SIV-infected macaques (224). Interestingly, CXCR5^+^ CD4^+^ T-cells also accumulate during chronic HIV infection (153, 223, 226), whereas this process is also driven by the local inflammation in SIV-infected macaques (227). Accordingly, increased immune activation and inflammatory cytokines secretion is present in lymph nodes from chronically HIV-infected patients (228). Interestingly, lymphotoxin α and β, and TNF are required for the expression of CXCL13 by murine follicular stromal cells (229), CXCL13 is expressed in chronic inflammation (230), and is a marker of chronic inflammation in HIV-infected patients, even during cART (231). Thus, the antigen burden in lymphoid tissues, both by infected CD4^+^ T-cells or virion-bearing follicular dendritic cells, may induce an inflammatory environment that in turn promote the production of CXCL13, contributing to the accumulation of CXCR5^+^ CD8^+^ T-cells.

Importantly, ART does not decrease the frequency of CXCR5^+^ CD8^+^ T-cells in human lymph nodes (222), which is in agreement with the low drug distribution to these tissues and consequent unchanged inflammatory environment, in comparison with blood (110). In addition, the compromise in follicles architecture during HIV infection may also be responsible for the increased passage of CD8^+^ T-cells and other cell types to these compartments (232).

**Figure 3:**
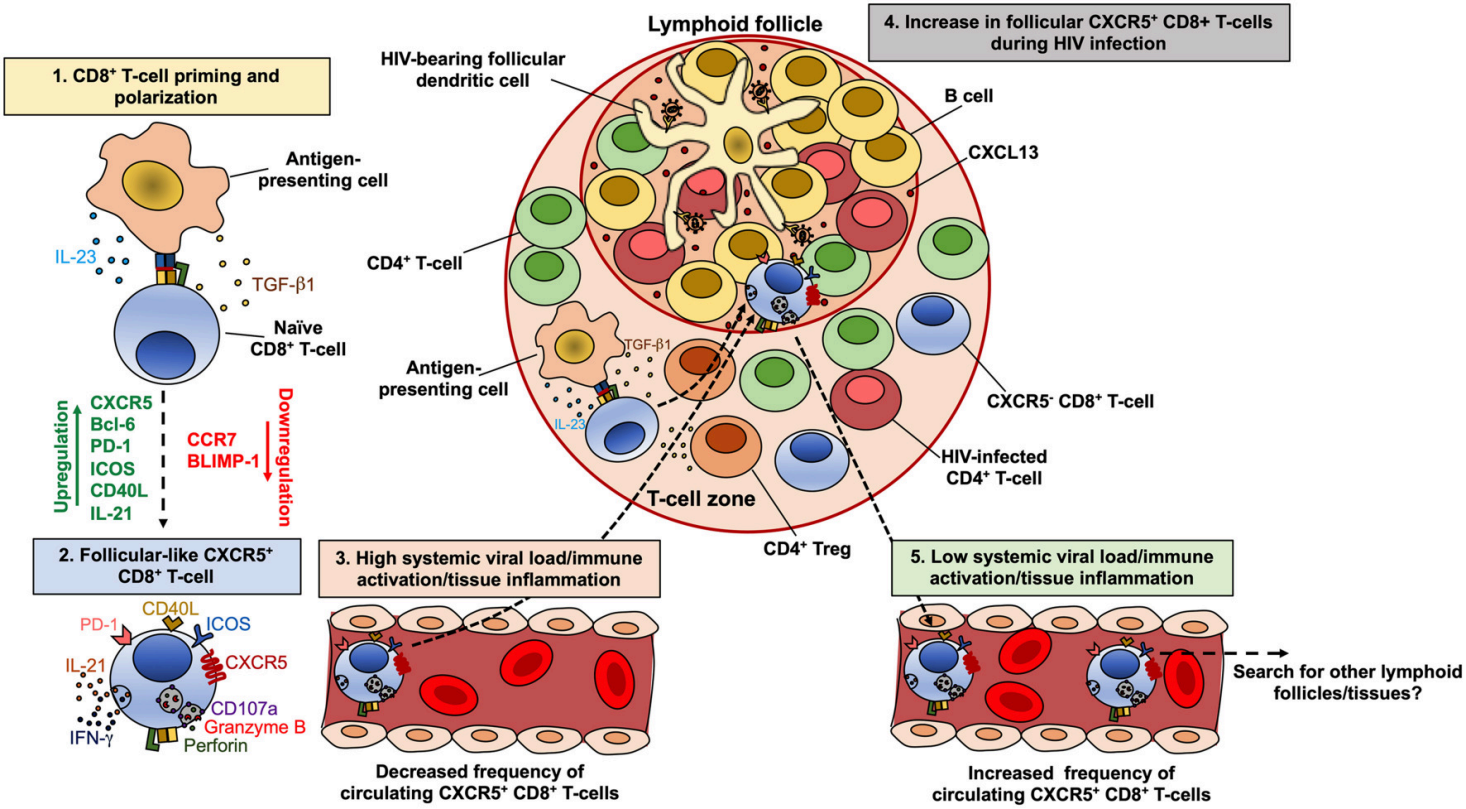
Model of the dynamics of CXCR5^+^ CD8^+^ T-cells during HIV infection. CD8^+^ T-cells are polarized to a follicular-like CXCR5^+^ profile after priming by an antigen-presenting cell that secrete TGF-β1 and IL-23 (1). These cytokines induce the up-regulation of CXCR5, Bcl-6, PD-1, ICOS, CD40L and IL-21, with down-regulation of CCR7 and BLIMP-1 (2). In circulation or peripheral tissues, follicular-like CXCR5^+^ CD8^+^ T-cells also exhibit a cytotoxic potential and production of IFN-γ. During chronic HIV infection and in the setting of high viral load and consequent increased immune activation and tissue inflammation, there is an accumulation of CXCR5^+^ CD8^+^ T-cells in lymphoid follicles, in response to CXCL13 (4). The high levels of TGF-β1 produced by antigen-presenting cells or regulatory T cells (Treg), may also induce the differentiation and accumulation of CXCR5^+^ CD8^+^ T-cells. Once in the follicles, CXCR5^+^ CD8^+^ T-cells exert an antiviral effect, which can be reflected in lower viral replication and the subsequent decrease in the immune activation/tissue inflammation and the egress of this subset from follicles. In circulation, CXCR5^+^ CD8^+^ T-cells possibly search for other lymphoid follicles or peripheral tissues (5).

On the other hand, circulating CXCR5-expressing CD8^+^ T-cells, which exhibit a transitional memory phenotype, are decreased in untreated HIV-infected patients compared with seronegative controls, but are maintained in patients under suppressive cART (233). Interestingly, the decrease in CXCR5^+^ CCR7^−^ CD8^+^ T-cells is associated with the increase in CXCR5^−^ CCR7^−^ cells, which are most likely an effector memory population. Moreover, the frequency of CXCR5^hi^ CCR7^−/lo^ CD8^+^ T-cells is inversely correlated with the level of systemic HIV replication in untreated HIV-infected patients, particularly in elite controllers (233). Strikingly, while similar correlations between the number of circulating human CXCR5^+^ CD8^+^ T-cells and systemic viral load have been reported (221), comparable associations were obtained between the frequencies of lymph node-confined memory CXCR5^+^ CD8^+^ T-cells and systemic viral load in HIV controllers patients (53). Thus, immune activation and local inflammation, but not viral replication, are apparently major drivers of CXCR5^+^ CD8^+^ T-cells (and CXCR5^+^ CD4^+^ T-cells) accumulation in lymphoid follicles during chronic HIV/SIV infection (Figure 3). Once in the lymphoid follicles, antigen-specific CXCR5^+^ CD8^+^ T-cells could partially eliminate infected cells, being reflected in lower local and systemic viral load, as has been observed in SIV-infected macaques (234). In fact, a role of this subpopulation in the control of HIV (70, 221) and SIV (225, 235) replication has been proposed, their antiviral function in follicles could be an important mechanism of disease protection, and its frequency could be as useful correlate of limited viral replication. If the systemic viral load, immune activation and/or tissue inflammation decrease, these cells could egress from the follicles, enter systemic circulation and search for other lymphoid follicles or tissues (Figure 3). However, it is yet unclear which other factors may influence the induction of CXCR5 and follicle migration of CD8^+^ T-cells.

In order to explore the differentiation conditions of human CXCR5^+^ CD8^+^ T-cells, we evaluated the effect of TGF-β1, IL-23, and IL-12, which promote the differentiation of human follicular CD4^+^ T-cells (236) and induce the expression of CXCR5 in non-human primate CD8^+^ T-cells (234). *In vitro*, TGF-β1 plus IL-23 (in the presence of TCR stimulation) induced the expression of CXCR5 in purified CD8^+^ T-cells from healthy individuals, as well as a follicular-like phenotypic and transcriptional profile, such as the up-regulation of PD-1, inducible T-cell costimulatory (ICOS), CD40L and *BCL6*, and the down-regulation of CCR7 and *PRDM1* (Perdomo-Celis F, In press). These data suggest that, *in vivo*, TGF-β1 and IL-23 may be polarizing factors for follicular-like CXCR5^+^ CD8^+^ T-cells, which could then migrate to lymphoid follicles for exerting immune surveillance (Figure 2). Considering the increased levels of regulatory T cells (Treg)-derived TGF-β1 in lymphoid tissues during HIV infection (237), there could be an appropriate environment for the differentiation and accumulation of follicular-like CXCR5^+^ CD8^+^ T-cells (Figure 3). Importantly, the induction of the expression of CXCR5 in CD8^+^ T-cells through cytokine stimulation or genetic engineering may be a useful strategy for improving the migration of these cells to lymphoid follicles and boost follicular antiviral responses that help to eradicate viral reservoirs in these structures (202, 238, 239).

## CD8^+^ T-Cell-Based Strategies To Treat Or Cure HIV Infection

Current strategies for CD8^+^ T-cell-based strategies to treat or cure HIV infection are based on stimulating pre-existing and/or inducing *de novo* HIV-specific immune responses through vaccine therapies or redirecting HIV-specific CD8^+^ T-cells and improving their function and persistence after adoptive transfer. The latter adoptive transfer strategies can be divided in (i) Expansion of HIV-specific CD8^+^ T-cells; (ii) Artificial CD8^+^ TCR; (iii) Chimeric antigen receptors (CAR); (iv) Selection of CD8^+^ T-cells with long-term persistence; (v) Induction of HIV-specific CD8^+^ T-cells from virus-naïve donor cells. Of note, these strategies may also be accompanied by the aforementioned immunomodulatory approaches, in order to reach a global reconstitution of the CD8^+^ T-cell response, as well as with latency-reversing agents, to induce the reactivation of viral reservoir (240), particularly in the case of patients receiving cART.

### CD8^+^ T-Cell-Based Vaccines

Several HIV vaccine candidates have been focused on the induction of CD8^+^ T-cell responses in order to control viremia, i.e., as a therapeutic approach. They range from whole attenuated virus, vector viruses containing HIV proteins, DNA plasmids, and HIV peptides [reviewed in (241)]. Importantly, immunization should be performed with multiple selected peptides based on conserved regions of HIV proteins, which induce subdominant but effective CD8^+^ T-cell responses, and are restricted by the most common HLA alleles in a specific population (242, 243). Nonetheless, in general, a low and transient reduction of viral load has been observed with most therapeutic vaccines (241), indicating that a combination of this and other approaches is required for a relevant impact on disease progression.

### HIV-Specific CD8^+^ T-Cells Adoptive Transfer

First reports including small cohorts of HIV-infected patients (some of them receiving cART) demonstrated that the infusion of autologous CD8^+^ T-cells enriched for HIV-specific cells targeting several viral proteins (expanded *ex vivo* through polyclonal stimulation), transiently decreased plasma viremia or productively infected cells, and increased CD4^+^ T-cell counts (244, 245). Nonetheless, in other studies, a rapid apoptosis of adoptively transferred HIV-specific CD8^+^ T-cells was associated with failure in the reduction of viral load (246). Moreover, transfer of HIV-specific CD8^+^ T-cells induced selection of escape mutant HIV variants, which was associated with rise in viral load and decrease of CD4^+^ T-cells (247). More recently, other approaches based on infusion of CD8^+^ T-cells specific for multiple HIV antigens (248) demonstrated efficacy in the control of autologous reservoir virus (249). Similarly, CD8^+^ T-cells transduced with vectors expressing a TCR targeting the relevant HIV epitope SL9 in Gag protein, showed lysis capacity and reduced infectious cells *in vivo* in SCID mice (250), as well as functional capacity in cells derived from cART-receiving patients (251). However, engineered TCR may have recognition of self-epitopes, resulting in severe reactions such as cardiac toxicity (252), which limits the use of this approach. The use of CAR CD8^+^ T-cells may overcome the problems of viral escape, HIV-induced HLA downregulation, and immune exhaustion (253). First-generation CAR T-cells containing the extracellular domain of human CD4 linked to the CD3ζ chain (CD4ζ) showed prolonged survival and trafficking to gut mucosa, although did not change viral load in HIV-infected patients (254). More recently, the CD4ζ CAR was re-engineered to express the 4-1BB costimulatory domain, inducing a more potent HIV suppressing capacity *in vitro* and *in vivo* in a humanized mouse model (255). CAR T-cells have also been designed with expression of single-chain variable fragments of broadly neutralizing antibodies (256), or carbohydrate-recognition domain of C-type lectin, both targeting gp120 (257). Another approach to improve the survival of adoptively transferred HIV-specific CD8^+^ T-cells is to select those with a long-term memory profile. Accordingly, *ex vivo* expanded HIV-specific CD28^+^ CD8^+^ T-cells survived for at least 84 days after infusion in cART-receiving patients and maintained central memory characteristics (258). Finally, functional HIV-specific CD8^+^ T-cells can be generated from HIV-negative donors through priming with peptide-pulsed dendritic cells, followed by polyclonal expansion. This approach could be useful in the setting of hematopoietic stem cell transplantation in HIV-infected patients to recover or improve the virus-specific CD8^+^ T-cell response (259).

In summary, these studies demonstrate the potential of CD8^+^ T-cell-based therapeutic strategies for HIV treatment or cure strategies. More importantly, combination of different strategies, such as vaccine induction of virus-specific CD8^+^ T-cells and subsequent adoptive transfer, could limit viral replication and/or rebound after cART interruption, as recently evidenced in a macaque model (260).

## Conclusions and Future Perspectives

Despite intense research on HIV, there is no preventing vaccine and cure available for this infection. In fact, a high incidence is still reported worldwide, underlying the importance of providing an optimal cART and health care management to HIV-infected patients, not only for improving their quality of life and preventing several co-morbidities, but also for breaking the chain of transmission. Thus, the characterization of HIV-infected patients under suppressive cART is required to understand the benefits, pitfalls, and challenges of cART, to identify novel immunomodulatory targets, and to elucidate potential strategies for reaching a functional or sterilizing cure for this infection. Despite complete suppression of viral load and reconstitution of CD4^+^ T-cells, HIV-infected patients exhibit increased systemic inflammation levels and a compromised CD8^+^ T-cells response, characterized by high activation/exhaustion and impaired lytic and non-lytic mechanisms. Some of the phenotypic and/or functional CD8^+^ T-cell features could be used in the clinical setting to identify those patients reaching immune reconstitution during continuous suppressive cART. Some of the CD8^+^ T-cell alterations, such as low IL-17 production, could be targeted by immunomodulatory drugs. Finally, follicular CXCR5^+^ CD8^+^ T-cells could be a therapeutic weapon for targeting viral reservoirs in lymphoid follicles, along with other CD8^+^ T-cell-based strategies.

## Author Contributions

FP-C, NT, and MR reviewed the literature and wrote the manuscript.

### Conflict of Interest Statement

The authors declare that the research was conducted in the absence of any commercial or financial relationships that could be construed as a potential conflict of interest.
